# Pharmacodynamic modeling of colistin and imipenem against *in vitro Pseudomonas aeruginosa* biofilms

**DOI:** 10.1016/j.bioflm.2026.100387

**Published:** 2026-08-03

**Authors:** Yuchen Guo, Jinqiu Yin, Linda B.S. Aulin, Oana Ciofu, Claus Moser, Parth J. Upadhyay, Niels Høiby, Hengzhuang Wang, Tingjie Guo, J.G. Coen van Hasselt

**Affiliations:** a Leiden Academic Centre for Drug Research, Leiden University, Leiden, the Netherlands; b Department of Clinical Microbiology, Rigshospitalet, Copenhagen, Denmark; c Department of Immunology and Microbiology, Costerton Biofilm Center, Faculty of Health Sciences, University of Copenhagen, Copenhagen, Denmark

**Keywords:** Biofilm, Pharmacodynamics, Modeling, *Pseudomonas aeruginosa*, Antibiotics

## Abstract

**Introduction:**

Antibiotic treatment of chronic biofilm-associated infections can be challenging. Characterization of pharmacokinetic/pharmacodynamic (PK/PD) relationships for biofilm-associated infections may be relevant to informing the design of antibiotic treatment regimens for biofilm-associated infections. To this end, we aim to develop a mathematical PK/PD model for planktonic and biofilm bacterial infections and demonstrate how PK/PD simulations can be used to design optimized dosing schedules, using colistin and imipenem as proof-of-concept examples.

**Methods:**

Pharmacodynamic models were developed using time-kill assay data from planktonic and alginate-bead biofilm cultures of *Pseudomonas aeruginosa* exposed to colistin or imipenem. The PD models were coupled to population PK models for plasma and lung epithelial lining fluid (ELF) to translate PD relationships for clinical dosing schedules and PK/PD indices.

**Results:**

The developed models incorporated susceptible and resistant bacterial subpopulations and were able to adequately capture the observed time-kill data. Simulation studies identified differences in suppression of bacterial growth dynamics for multiple clinical intravenous and inhalation-based treatment regimens and were used to infer biofilm-specific PK/PD indices associated with ELF target site concentrations.

**Conclusion:**

In conclusion, we demonstrate the utility of mathematical modeling for the characterization of PK/PD relationships underlying time-kill kinetic profiles in biofilm-associated infections and their utility in translating experimental findings toward clinically relevant dose-optimization strategies.

## 1 Introduction

Chronic lung infections in cystic fibrosis (CF) patients are typically associated with bacterial biofilms and respond poorly to antibiotic therapy [[1], [2], [3]]. Management often involves long-term antibiotic therapy, including daily nebulized antibiotic treatment and systemic antibiotic treatment during acute exacerbations [4,5]. Biofilm-associated pathogens often show reduced antibiotic sensitivity compared to their planktonic form, mediated by multiple mechanisms [6]. In addition, the antimicrobial concentrations at the target-site, i.e., the lungs in case of chronic CF lung infections, can differ substantially from plasma concentrations [7,8]. There is a need to further optimize antibiotic dosing schedules for the treatment of biofilm-associated chronic lung infections in CF patients.

A rational treatment design for biofilm-associated bacterial infections requires information on the antibiotic concentration-time profile at the site of infection (pharmacokinetics, PK) and the observed relationship between drug exposure and response of bacterial pathogens (pharmacodynamics, PD). In terms of PD, different mechanisms contribute to the decreased susceptibility of biofilm bacteria [9]. For example, the formation of extracellular matrix protects the bacteria embedded within it from the attack of the immune system and poses a diffusion barrier against antibiotics. In addition, bacterial pathogens may develop resilience against antibiotic treatment mediated through transient adaptation or non-transient genetic mutations.

Antimicrobial PK/PD relationships can be characterized using experimental *in vitro* and *in vivo* models. Static *in vitro* assays, such as minimum inhibitory concentration (MIC) or minimum biofilm inhibitory concentration (MBIC), could provide rapid insights into antimicrobial susceptibility, yet they are measured at a single time point (e.g., 24 h) and do not capture dynamic responses, for example, the emergence of transient or persistent antimicrobial resistance [10]. In contrast, time-kill assays conducted *in vitro* allow characterization of the full time course of bacterial responses to antimicrobial agents [[11], [12], [13]], providing essential information about pathogen-associated PD relationships.

Mathematical mechanism-based PD models are useful tools in quantitatively characterizing bacterial growth and kill dynamics determined by time-kill assays. Such mechanism-based PD models support systematic testing of hypotheses that may explain observed pharmacodynamic responses with respect to delays (e.g.*,* due to drug diffusion into the biofilm), differences in growth rates of bacterial subpopulations, and concentration-effect relationships [14]. More importantly, when PD models are coupled to population PK models that predict antimicrobial concentration-time profiles in patients [13,15,16], the efficacy of clinical dosing schedules can be evaluated to assess alternative optimal dosing regimens. Most antimicrobial PK/PD models have focused on planktonic bacterial pathogens, lacking attention to biofilm-associated pathogens [17]. To optimize the dosing schedules for biofilm-associated infections, it is essential to quantitatively characterize their PK/PD relationships.

In this study, we aim to demonstrate the utility of mathematical PK/PD modeling for analyzing experimental biofilm time-kill studies, with the goal of characterizing pharmacodynamic differences between bacterial pathogens in biofilm versus planktonic lifestyles. As a proof of concept, we focus on colistin and imipenem for the treatment of the CF-associated pathogen *Pseudomonas aeruginosa*. Specifically, our objectives are to: (1) develop PD models for colistin and imipenem using data from *in vitro* time-kill studies in planktonic cultures and alginate-bead biofilm experiments [18,19]; and (2) integrate the developed PD models with population PK models for plasma and lung concentrations to explore expected clinical PK/PD profiles and indices, and to evaluate how PK/PD relationships may be altered under biofilm conditions.

## 2 Methods

### 2.1 Time-kill studies

Previously published time-kill studies of *P. aeruginosa* PAO1 planktonic [18] and alginate bead biofilm cultures [19] exposed to colistin or imipenem were used for mathematical model development (Fig. S1). Briefly, the inoculum for both planktonic and alginate bead experiments was 10^6^ CFU/mL in lysogeny broth medium. Beads (50-100 μm) were produced by embedding *P. aeruginosa* in seaweed alginate [19]. For colistin, both planktonic and alginate bead biofilm cultures were exposed to colistin at concentrations of 0-256 mg/L for 24 h. For imipenem, planktonic cultures were tested against imipenem at concentrations of 0-32 mg/L for 24 h, while additional concentrations up to 2048 mg/L were included for the alginate bead biofilm experiments. The studied concentrations covered a relatively wide efficacy range. Samples were taken for CFU quantification at 0, 1, 2, 4, 8, 12 and 24 h post antibiotic exposure (Table S1).

### 2.2 Mathematical model development

Ordinary differential equation (ODE)-based compartmental models were developed to describe the bacterial growth and kill dynamics in planktonic and biofilm cultures. The models included subpopulations of susceptible (S) and resistant (R) bacteria, where resistant bacteria were assumed to reflect a bacterial subpopulation with reduced sensitivity. Colony forming units (CFU) data were log_10_-transformed prior to the analysis. Models for planktonic and biofilm bacteria were developed separately. Log-transformed observed data were used to estimate the parameters that maximized the log-likelihood.

We incorporated natural growth kinetics, the net growth of bacteria in absence of antibiotics, for planktonic and biofilm cultures. A capacity-limited growth model was used (Eq. (1)), including parameters for the maximum bacterial density ($B_{\max}$), and a first-order net growth rate ($k_{gs}$), with a starting bacterial density (CFU/mL) of *B*_*0*_.

(1) $$\frac{dS}{dt}=\left(1-\frac{S}{B_{\max}}\right)\cdot k_{gs}\cdot S$$

Colistin and imipenem exhibit significant loss during *in vitro* experiments. To accurately define PK/PD relationships, colistin and imipenem concentration-time profiles were simulated, taking into account colistin degradation and plastic binding, as well as imipenem degradation, during the time-kill assay (Fig. S2). The *in vitro* PK model for colistin was fitted using literature data [20], while the degradation of imipenem was modeled with a 1st order elimination rate based on the literature [21,22] (Supplementary material).

Drug effect is reflected in the killing rate constant of bacteria, which describes the rate at which bacteria are killed per time. Drug concentration-effect functions evaluated included linear (Eq. (2)), power (Eq. (3)) and Emax functions (Eq. (4)), separately, for each individual subpopulation (i.e., S, R). Antibiotic concentration-effect models (i.e., linear, power or Emax) were defined as follows:

(2) $$E_{drug}=Slope\cdot C_{drug}$$

(3) $$E_{drug}=Slope\cdot {C_{drug}}^{power}$$

(4) $$E_{drug}=\frac{E_{\max}\cdot C_{drug}}{{EC}_{50}+C_{drug}}$$

where Slope is the linear kill effect coefficient; power represents the exponent for drug effect; $E_{\max}$ represents the maximum kill rate constant, ${EC}_{50}$ indicates the drug concentration at which 50% of the maximum killing rate is obtained.

We investigated the occurrence of an effect delay in biofilm cultures, potentially resulting from retarded drug diffusion into the biofilm. Such a delay would account for a possible discrepancy between the experimentally exposed drug concentration ($C_{{drug}_{\exp}}$) and the effective drug concentration that exerted pharmacodynamic effect on bacteria. The delay was described using a transit model (Eq. (5)), with a first-order transit rate constant $k_{tr}$, including *n* transit compartments. The concentration in the last transit compartment (${C_{drug}}_{\left(n\right)}$) represented the concentration driving the effect. Mean transit time (*MTT*), the average time spent by drugs traveling from the first transit compartment to the last compartment, was calculated with $k_{tr}$ and *n* (Eq. (6)).

(5) $$\begin{array}{c} \frac{dC_{{drug}_{\left(1\right)}}}{dt}=k_{tr}\cdot C_{{drug}_{\exp}}-k_{tr}\cdot C_{{drug}_{1}}\;\\ \ldots \\ \frac{d{C_{drug}}_{\left(n\right)}}{dt}=k_{tr}*{C_{drug}}_{\left(n-1\right)}-k_{tr}*{C_{drug}}_{\left(n\right)} \end{array}$$

(6) $$MTT=\frac{n+1}{k_{tr}}$$

We incorporated a drug-induced transition rate $k_{sr}$ to describe the transfer of bacteria from S to R state, which only occurred if an antibiotic is present (Eqs. (7) and (8)), for both planktonic and biofilm bacteria. Initially, all bacteria were assumed to be in a susceptible (S) state. First-order growth rates for S and R populations were estimated separately. Drug-induced killing effect was described using a first-order rate process for each subpopulation separately.

(7) $$\frac{dS}{dt}=\left(1-\frac{S+R}{B_{\max}}\right)\cdot k_{gs}\cdot S-k_{sr}\cdot S-E_{drug,S}\cdot S\;;\;S\left(0\right)=B_{0}$$

(8) $$\frac{dR}{dt}=\left(1-\frac{S+R}{B_{\max}}\right)\cdot k_{gr}\cdot R+k_{sr}\cdot S-E_{drug,R}\cdot R\;;\;R\left(0\right)=0$$

Mathematical models were first developed using pooled control group data from the colistin and imipenem experiments to capture the natural growth kinetics of planktonic and biofilm bacteria. Then PD models of colistin and imipenem were developed based on the treatment group data while fixing the natural growth kinetic-associated parameters for the susceptible subpopulation. Since drug concentration-effect may vary across drugs, bacterial subpopulations and lifestyles, separate modeling was carried out for each set of experiment. An additive error model for log-transformed data was used to estimate residual unexplained variability. Bacterial counts below the lower limit of quantitation (LLOQ, defined as 10 CFU/mL) were handled using the M3 method [23]. The final model was evaluated and selected based on successful convergence, reasonable parameter estimates and precision, goodness-of-fit (GOF) plots, and visual predictive checks.

### 2.3 Sensitivity analysis

To determine the relative importance of model parameters, a sensitivity analysis was performed for each parameter (*p*) in the final model. For each drug concentration, the local sensitivity Sens was evaluated using the relative change in the area under bacterial time-kill curve (AUBC) between 0 and 24 h, in relation to the relative change of parameters (Eq. (9)) [24].

(9) $$Sens=\frac{\Delta AUBC}{AUBC}÷\frac{\Delta p}{p}$$

### 2.4 Dosing regimen simulations

Published population PK models of colistin and imipenem [25,26] were used to predict concentration-time profiles in plasma and lung epithelial lining fluid (ELF), which were then integrated with the PD model to simulate bacterial response under humanized ELF PK profiles. For colistin, we studied intravenous administration of 160 mg (2 MIU) every 8 h of colistimethate sodium (CMS), the inactive prodrug of colistin, 720 mg (9 MIU) CMS every 24 h, and inhalation of 160 mg (2 MIU) CMS every 8 h, consistent with recommended clinical dosing regimens [27]. For imipenem, we simulated clinical tolerable doses [4]: 250, 500 and 1000 mg every 6 h intravenously. The 1000 mg dose represents an extrapolation from the imipenem PK model, but given the linear PK properties reported in other studies and its clinical relevance, this dose was included. For each dosing regimen, 1000 individuals were simulated. All intravenous infusions were administered over 30 min, except for the 1000 mg imipenem infusion, which was infused over 60 min. Interindividual variability was incorporated in both colistin and imipenem simulations. For imipenem, in addition to interindividual variability, we incorporated the covariate effects of body weight and creatinine clearance. As these covariates were often correlated, a virtual population was generated using the NHANES copula (https://cocosim.lacdr.leidenuniv.nl/) to obtain realistic covariate data for PK simulation results [28,29]. The simulated ELF profiles were linked to PD models for planktonic and biofilm bacteria to assess the relative difference in bacterial dynamics under different dosing schedules for planktonic and biofilm-associated infections. Given the low protein concentrations in ELF relative to plasma, colistin and imipenem in this compartment was assumed to be fully unbound (free fraction = 100%) and pharmacologically active [26,30,31].

### 2.5 PK/PD target analyses

To identify PK/PD indices for colistin and imipenem against planktonic and biofilm bacteria, we performed extensive dose fractionation simulations across a wide range of doses and dosing intervals, following the approach of a previous study [32]. For each dosing schedule, we computed the PK/PD indices, including the maximum ELF free concentration of drug over the minimum inhibitory concentration (fC_max_/MIC) and over the minimum biofilm inhibitory concentration (fC_max_/MBIC), area under the concentration-time curve for drug over the MIC (fAUC/MIC) and over the MBIC (fAUC/MBIC), and the fraction of time when the free concentration was above the MIC (f_T > MIC_) and above the MBIC (f_T > MBIC_). We used the MIC and MBIC values obtained from the same strain used in the time-kill experiments. The MIC and MBIC values were 2 and 8 mg/L for colistin, and 1 and 8 mg/L for imipenem, respectively [18]. The PK profiles were used to predict the treatment response in planktonic and biofilm bacterial infections using the established PD models. For each bacterial lifestyle against each drug, we regressed PK/PD indices against the change of bacterial density (log10 CFU/mL) after 24 h of treatment using a sigmoidal E_max_ equation. Although alternative functions could also describe the exposure–response relationships, we selected the sigmoidal Emax function because it is widely used in antimicrobial PK/PD target analyses [13] and ensures consistency across all bacterial lifestyle - antibiotic groups. The model fit was evaluated using the R^2^ value to identify the PK/PD indices that best predicted bacterial killing after 24 h (e.g.-1 and −2 log10 kill).

### 2.6 Software and model selection

Model development was performed using non-linear mixed fixed effects modeling software NONMEM 7.5. Graphical visualizations, simulations and interpretations were performed using R 4.3.2. Model selection was guided by successful minimization, successful covariance step, objective function value, Akaike information criterion, precision of parameter estimates, goodness-of-fit (GOF) plots, and visual predictive checks [33].

## 3 Results

### 3.1 Mathematical model development

**Fig. 1:**
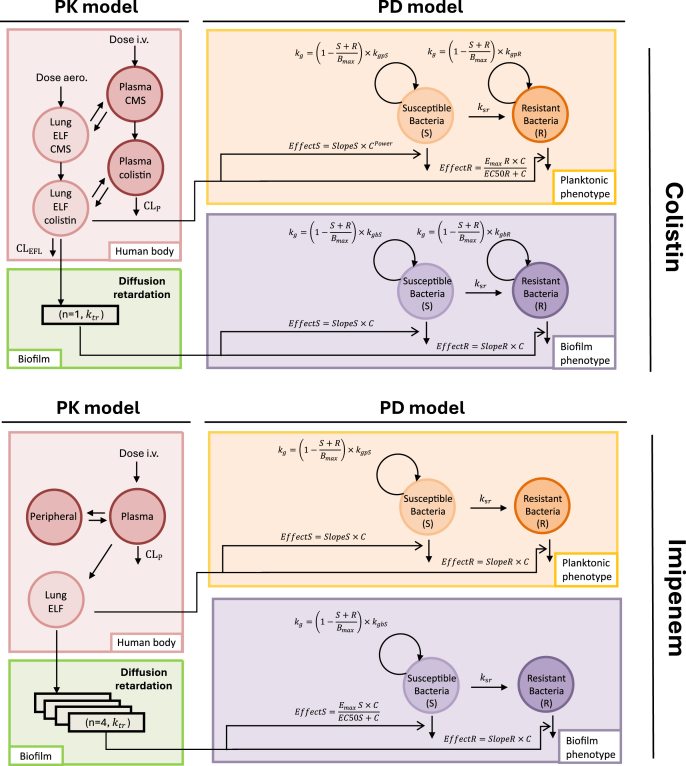
**Schematic illustration of the pharmacokinetic-pharmacodynamic model structures.** Model structures for colistin and imipenem included the pharmacodynamic models describing the time-kill kinetics in planktonic and alginate bead biofilm models, in combination with clinical pharmacokinetic models for plasma and lung epithelial lining fluid (ELF) compartments. Separate pharmacodynamic models were developed for planktonic and biofilm bacteria cells for colistin and imipenem.

**Fig. 2:**
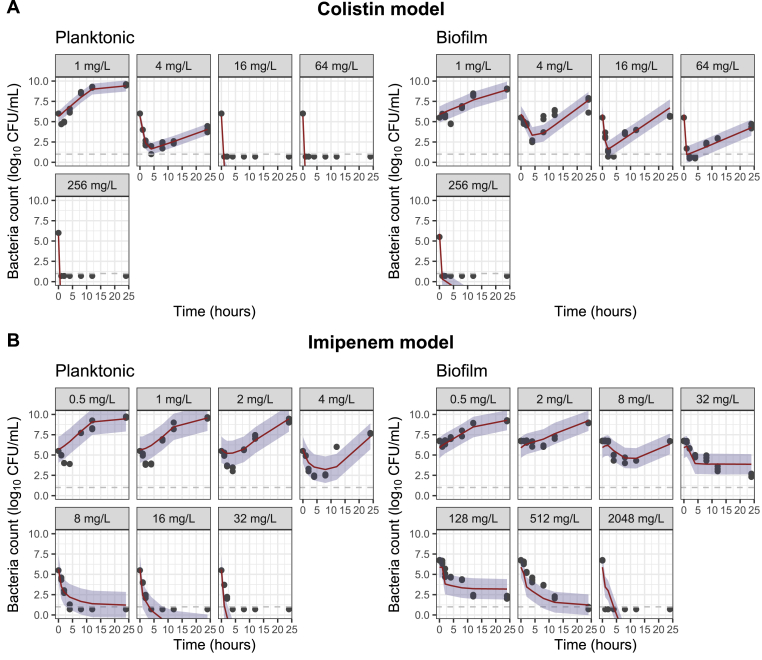
**Visual predictive check of the pharmacodynamic models for colistin (A) and imipenem (B).** The black points are the observed bacterial count data (Log10 CFU); the red lines represent the median values of model predictions; The blue areas are the prediction interval defined by of the 10th to 90th percentiles of the model predictions. Observations below the quantification limit (gray dashed line) were displayed as half of the quantification limit. (For interpretation of the references to colour in this figure legend, the reader is referred to the Web version of this article.)

Natural growth kinetics of planktonic and alginate bead biofilm bacteria were well characterized (Fig. S3–S5). Both colistin and imipenem showed a concentration-dependent killing effect against planktonic and biofilm-embedded *P. aeruginosa*. Regrowth was observed after approximately 4 h in both bacteria when exposed to colistin and 4-8 h when exposed to imipenem (Fig. S1). The time-kill kinetics of planktonic and biofilm cultures exposed to antibiotics were described separately using pharmacodynamic models (Fig. 1). The models adequately captured the observed bacterial growth- and kill-profiles (Fig. 2), with the adequate fit further demonstrated by the GOF plots (Fig. S6–S9). Most model parameters were estimated precisely, with relative standard errors ≤ 30% (Table 1). EC50R in the colistin-planktonic model had a slightly higher RSE (37%), likely due to the limited coverage of low drug concentrations in the experimental design.

**Table 1:** Parameter estimates of the pharmacodynamic model for colistin and imipenem for *in vitro* planktonic and biofilm time-kill assays.

Parameter	Description	Units	Planktonic (RSE%)		Biofilm (RSE%)	
			Colistin	Imipenem	Colistin	Imipenem
$k_{gs}$	Maximal growth rate of susceptible bacteria	h^−1^	0.9 (8)^a^		0.572 (8)^a^	
$k_{gr}$	Maximal growth rate of resistant bacteria	h^−1^	0.9^b^	0^c^	0.6 (5)	0^c^
$B_{0}$	Baseline bacterial count	log_10_ CFU/mL	5.67 (2)^a^		5.9 (2)^a^	
$B_{\max}$	Bacterial count in stationary phase	log_10_ CFU/mL	9.52 (1)^a^		9.24 (2)^a^	
$k_{sr}$	Transfer rate from Susceptible bacteria to resistant bacteria	-log_10_ h^−1^	3.79 (9)	1.46 (8)	4.14 (4)	2.3 (7)
SlopeS	Linear coefficient for drug effect in susceptible bacteria	L/mg*h	1.21 (11)	0.857 (4)	0.831 (3)	-
PowerS	Exponent for drug effect in susceptible bacteria	-	1.39 (8)	-	-	-
EmaxS	Maximal kill rate constant in susceptible bacteria	h^−1^	-	-	-	54.6 (16)
EC50S	Concentration that results in 50% of Emax for susceptible bacteria	mg/L	-	-	-	170 (12)
SlopeR	Linear coefficient for drug effect in resistant bacteria	L/mg*h	-	0.135 (4)	0.0054 (6)	0.0021 (14)
EmaxR	Maximal kill rate constant in resistant bacteria	h^−1^	9.33 (30)	-	-	-
EC50R	Concentration that results in 50% of Emax for resistant bacteria	mg/L	35.1 (37)	-	-	-
$k_{tr}$	Distribution rate constant between transit compartments	h^−1^	-	-	4.27 (27)	1.57 (9)
N	Number of transit compartments	-	-	-	1	4
RES	Additive residual error in bacteria experiment (log10 scale)	-	0.537 (10)	1.25 (7)	0.788 (8)	0.977 (6)

RSE, relative standard error; RES, residual variance error.

a Natural bacteria growth parameters were estimated using pooled control group data from the colistin and imipenem experiments.

b The same growth rates for resistant and susceptible bacteria were assumed.

c The value of the parameter converged to 0 and was therefore fixed as 0.

The growth rate constant for resistant populations in imipenem experiment converged to 0 and was therefore fixed at 0. This suggested the emergence of non-growing cells upon the exposure of imipenem. We identified linear relationships between drug concentration and effect in all models except for colistin planktonic model. For colistin planktonic model and imipenem biofilm model, Emax models best described the relationship for the resistant and susceptible population, respectively, while the power function best described the relationship for susceptible population in colistin planktonic model.

For both colistin and imipenem, we estimated the drug-specific rate of diffusion retardation into the biofilm. We evaluated models with 1 to 4 transit compartments, and found 1 and 4 transit compartment(s) fitted the colistin and imipenem data best. Parameter estimates ($k_{tr}$ and *n*) revealed that imipenem exhibited a longer MTT (3.2 h) compared to colistin (0.5 h). Local sensitivity analysis identified the sensitive parameters that driving the predicted response for each model, quantified as the relative change in the area under the bacterial time-kill curve following a 10% change in each parameter. SlopeR showed large absolute sensitivity values in the colistin biofilm model and both imipenem models, indicating wide influence across both studied drugs (Fig. S10). In the colistin planktonic model, SlopeR was not applicable because the drug effect on the resistant subpopulation was described using an Emax model rather than a linear model.

### 3.2 Dosing regimen simulations

**Fig. 3:**
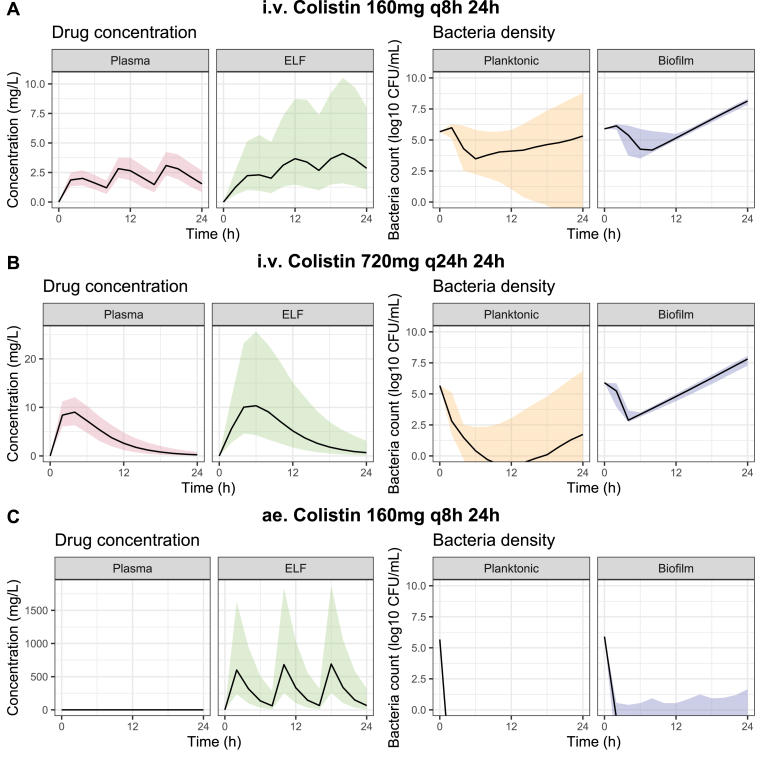
**Dosing regimen simulations for colistin.** Dosing regimens were simulated using clinical population pharmacokinetic models depicting the median (lines) and 25th and 75th percentiles (shaded areas) of drug concentration and bacteria count versus time. The predicted epithelial lining fluid (ELF) concentration was used as input for the pharmacodynamic models. Dosing regimens simulated included intravenous (i.v.) administration of 160 mg (2 MIU) CMS over 30 min every 8 h (**A**), 720 mg (9 MIU) CMS over 30 min every 24 h(**B**), or aerosol (ae.) inhalation of 160 mg (2 MIU) CMS every 8 h (**C**).

**Fig. 4:**
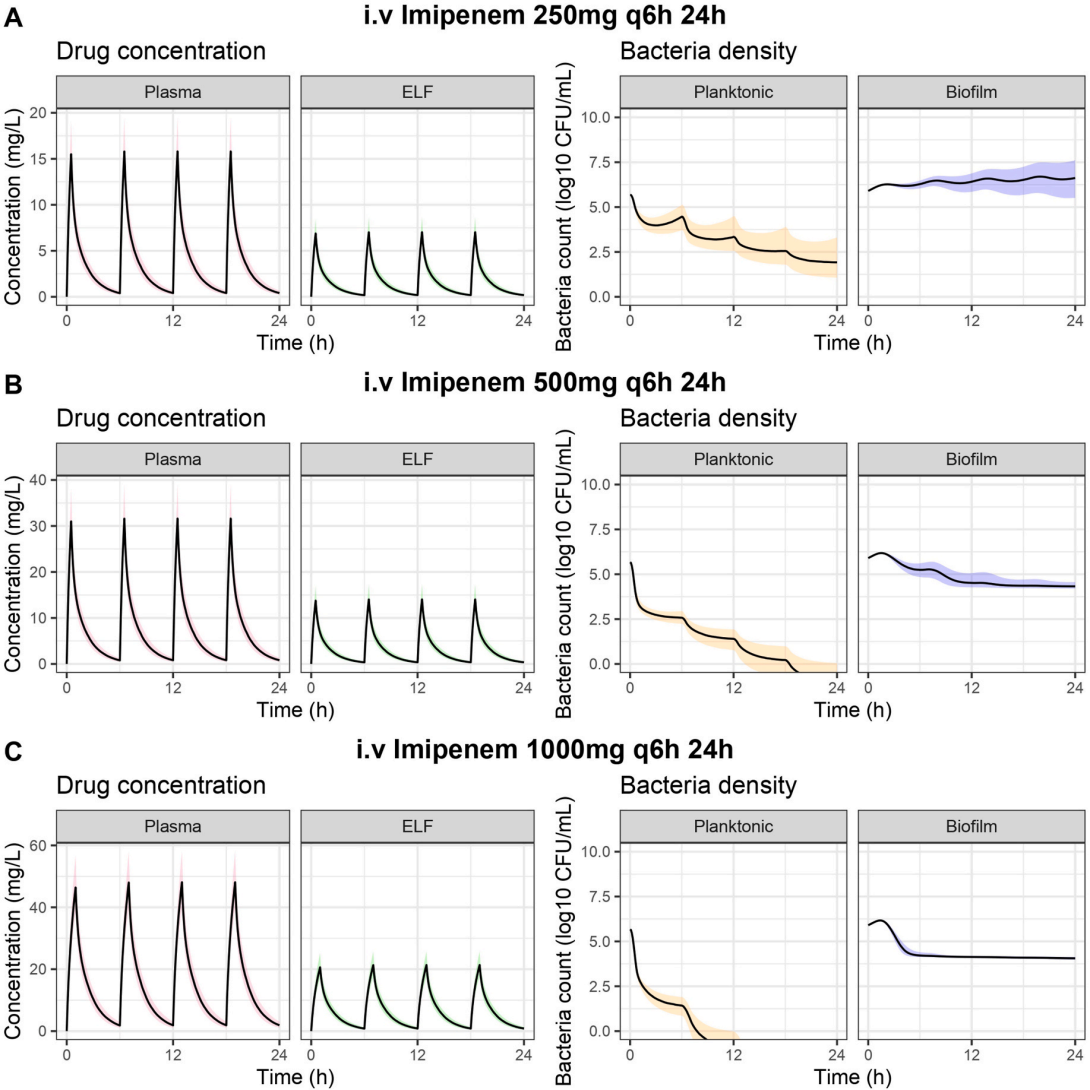
**Dosing regimen simulations for imipenem.** Dosing regimens were simulated using clinical population pharmacokinetic models depicting the median (lines) and 25 and 75th percentiles (shaded areas) of drug concentration and bacteria count versus time. The predicted epithelial lining fluid (ELF) concentration was used as input for the final pharmacodynamic models. Dosing regimens simulated included intravenous (i.v.) infusions of 250 mg over 30 min (**A**), 500 mg over 30 min (**B**), and 1000 mg over 60 min (**C**), every 6 h.

We simulated the treatment outcome of standard and adapted dosing schedules of colistin (Fig. 3) and imipenem (Fig. 4) against planktonic and biofilm-associated *P. aeruginosa* lung infections, using the PK/PD models (i.e., developed PD models coupled with clinical population PK models).

For colistin, our simulations showed that under the treatment of 160 mg (2 MIU) CMS every 8 h (Fig. 3A) and 720 mg (9 MIU) CMS every 24 h (Fig. 3B), colistin was insufficient for patients with biofilm infections. An inhalation dose of 160 mg (2 MIU) CMS resulted in a high drug concentration at the ELF, leading to a successful eradication of both planktonic and biofilm bacteria (Fig. 3C). For imipenem, a clear dose-dependent killing effect was found (Fig. 4). For planktonic bacterial infection, both 500 mg and 1000 mg every 6 h could kill planktonic bacteria efficiently. All treatments could suppress the growth of biofilm-associated cells yet were unable to fully eliminate the infections within 24 h.

### 3.3 PK/PD indices

**Fig. 5:**
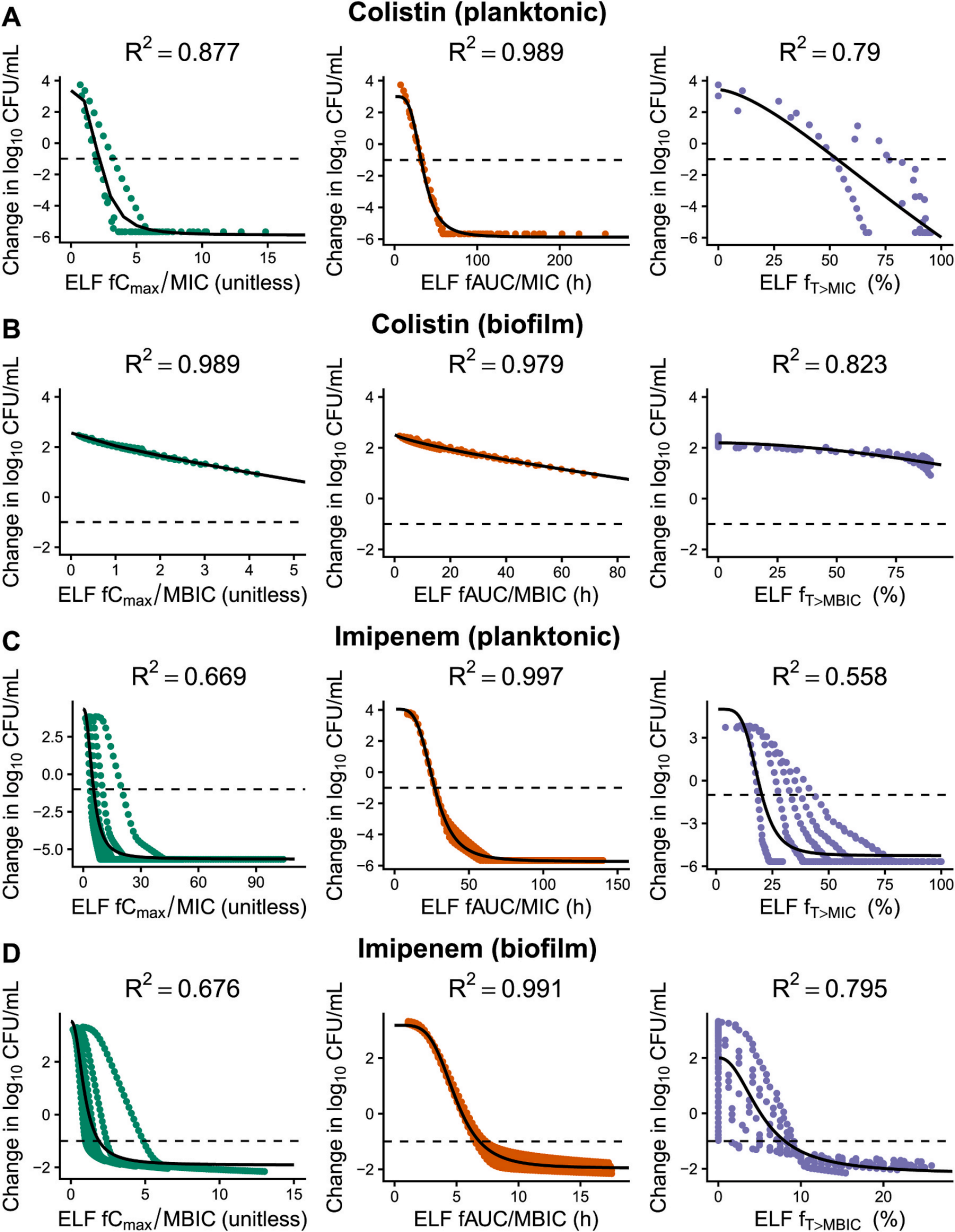
**Pharmacokinetic/pharmacodynamic (PK/PD) target analysis for colistin and imipenem against planktonic and biofilm infections based on epithelial lining fluid (ELF) antibiotic concentrations.** Dose fractionation studies were simulated across a range of total daily doses and dosing intervals. The resulting ELF concentration–based PK/PD indices were correlated with the model-predicted change in bacterial density at 24 h relative to baseline, with points representing each simulation and solid lines representing sigmoidal Emax model fits.

We investigated PK/PD indices for predicting treatment responses in planktonic and biofilm bacterial infections by performing intravenous dose fractionation studies based on the target site drug concentration in the ELF (Fig. 5). Across both drugs, fAUC/MIC was the best predictor of antimicrobial effect in planktonic infections (R^2^ = 0.989 for colistin; R^2^ = 0.997 for imipenem), whereas for biofilm infections, fCmax/MBIC (colistin, R^2^ = 0.989) and fAUC/MBIC (imipenem, R^2^ = 0.991) provided the strongest correlations. For planktonic bacteria, we found that for a CFU change from −1 log_10_ to −2 log_10_, the fAUC/MIC target for colistin and imipenem increased from 33 to 37, and from 27 to 30, respectively. For biofilm bacteria, a CFU change of −1 log_10_ and -2 log_10_ at 24 h corresponded to 12.1 and 16.1 for the fCmax/MBIC target of colistin. A −1 log_10_ change related to an fAUC/MBIC target of 6.9 for imipenem. However, no target of AUC/MBIC for imipenem was able to be derived for −2 log_10_ CFU change due to insufficient drug effect at tolerable intravenous dosages (Table 2). The treatment outcome under simulated dosing regimens aligned with the expected response based on the derived targets. For example, for biofilm infection, 160 mg (2 MIU) CMS every 8 h and 720 mg (9 MIU) CMS every 24 h (fCmax/MIC = 0.5, 1.6) did not achieve the PK/PD target and failed to kill the bacteria, while inhaled 160 mg (2 MIU) CMS every 8 h (fCmax/MIC = 110) reached the targets and killed the bacteria efficiently.

**Table 2:** ELF-based PK/PD target values derived for colistin and imipenem at −1 and −2 log10 unit kill using simulated dose fractionation studies.

	Colistin		Imipenem	
	Planktonic	Biofilm	Planktonic	Biofilm
	fAUC/MIC	fCmax/MBIC	fAUC/MIC	fAUC/MBIC
**−1 log10**	33	12.1	27	6.9
**−2 log10**	37	16.1	30	-

In terms of PK/PD indices based on plasma drug concentration, fAUC/MIC (R^2^ = 0.987) and fAUC/MBIC (R^2^ = 0.985) were best correlated with the change of bacteria count for colistin, while fAUC/MIC (R^2^ = 0.997) and fAUC/MBIC (R^2^ = 0.991) were still most predictive of the effect for imipenem (Fig. S11). The ELF-based (Fig. 5) and plasma-based (Fig. S11) PK/PD index analyses demonstrated consistent correlations between PK/PD indices and treatment effects for colistin and imipenem.

## 4 Discussion

We developed a pharmacodynamic model to characterize growth and kill dynamics from *in vitro* biofilm assays, focusing on the pathogen *P. aeruginosa* treated with colistin or imipenem, as a proof-of-concept. Prior knowledge of patient-specific antibiotic PK was integrated into the modeling framework and applied to investigate the effect of different dosing regimens, and the predictivity of different drug exposures (PK/PD indices) for bacterial response was assessed in clinical settings.

The alginate bead biofilm model used in this study is a well-established *in vitro* model that replicates key features of *P. aeruginosa* biofilms observed in chronic infections, and has been shown to display *in vivo* like characteristics [[34], [35], [36]]. Clinical isolates of *P. aeruginosa* might yield different results from the lab strain PAO1 used in this study, however, our primary objective is to demonstrate the application of mathematical models to enhance our understanding of resistance development and diverse bacterial lifestyles. This modeling framework also provides valuable insights into bacterial responses under clinical dosing regimens.

We assumed no resistant bacteria at the beginning of experiments for the initial population of bacterial cells, i.e., we did not consider the potential role of heteroresistance. It should be noted, however, that models which incorporate low-frequency pre-existing resistant population of cells versus de novo resistance/tolerance are typically mathematically indistinguishable if only total CFU data is available.

In this study, we derived models for different drugs, i.e., colistin and imipenem, as well as different bacterial lifestyles, i.e., planktonic bacteria and biofilm-associated bacteria. This model-based data-driven strategy enabled the evaluation of hypotheses with respect to differences in pharmacodynamic responses of planktonic versus biofilm-associated bacteria. The resulting models allowed the comparison of drug-, biological system- or experiment-specific parameters that ultimately characterize the observed response, providing insight into the underlying pharmacological basis of the antibiotic response. We identified distinct factors that contribute to increased resistance of biofilm-associated cells compared to planktonic cells against both colistin and imipenem. First of all, biofilm-associated cells exhibited slower growth rates (0.572 h^−1^) compared to planktonic cells (0.9 h^−1^), consistent with the literature [37,38]. This reduced growth rate reflects biofilm-associated cells’ adaptation to environments with limited nutrients and oxygen, which requires lower metabolic activity for survival. Secondly, a delay in antibiotic drug effect for biofilms, as represented by a transit model, was found for both colistin and imipenem against biofilm bacterial infections. This delay can be explained by the diffusion barrier presented by the extracellular matrix secreted by biofilm-associated bacteria. For example, the negatively charged polysaccharides could bind to positively charged colistin and impede the penetration into biofilm [39]. The alginate bead model used in this study has similarly been reported to interact with cationic antibiotics such as colistin and reduce the antibacterial activity compared to neutral polysaccharide biofilm models using agar [40]. Given the short transit time colistin (MTT = 0.5 h), additional sampling before 1 h would help to more precisely characterize this early phase or delay in effect. The slower transit of imipenem (MTT = 3.2h) is likely related to the fact that imipenem has been reported to induce β-lactamase production in *Pseudomonas aeruginosa* biofilms [41], with spatially heterogeneous expression across the biofilm [42]. This may contribute to local drug inactivation and an apparently extended transit time. Thirdly, compared to planktonic resistant subpopulations, a reduced susceptibility of biofilm resistant subpopulations was found (Table 1). This discrepancy might be relevant to further physiological adaptations in biofilm-associated bacteria compared to planktonic bacteria [43].

The developed PD models offered insights into resistance development under colistin and imipenem exposure. Colistin-treated bacteria displayed slower transfer rates (10^−3.79^ and 10^−4.14^ h^−1^) from susceptible to the resistant bacteria compared to imipenem-treated bacteria (10^−1.46^ and 10^−2.3^ h^−1^)*.* Regrowth under colistin exposure likely reflects adaptive modifications of the bacterial outer membrane, such as LPS alterations that reduce colistin penetration [[44], [45], [46]]. Identical growth rates were assumed for the susceptible and resistant subpopulations in colistin-treated planktonic bacteria. This is due to the identifiability issue arising from insufficient data specifically designed to capture bacterial regrowth. In imipenem-treated bacteria, the absence of growth in resistant subpopulations indicates rapid phenotypic adaptation and persister formation, likely driven by increased alginate and AmpC β-lactamase expression [[47], [48], [49]].

Coupling PD models with clinical population PK models enabled us to make translational predictions about the expected effects of bacterial growth/kill dynamics in patients. In PK/PD simulations for lung infections, ELF was assumed as a relevant lung compartment where antibiotics exert bacterial killing effects, with bacteria load in ELF representing infection dynamics. We recognize that antibiotic and bacterial distribution in the lung can be spatially heterogeneous, and that ELF concentrations may not accurately represent antibiotic levels in the interstitial space, where infection can occur. Given that this work is primarily demonstrative, ELF concentrations were used to illustrate and compare the predicted drug effects across agents and bacterial lifestyles, and potential alterations in free concentrations associated with infection were not considered [50]. For the efficacy of colistin, we predicted that dosing of 2 MIU per inhalation q 8 h shows a better biofilm-eradicating effect in lung infection patients compared to intravenous treatment of 2 MIU q 8 h and 9 MIU q 24 h. This result is in line with previous studies in COPD patients and ICU patients with pulmonary infections, where nebulized colistin was associated with improved outcomes [51,52]. Imipenem showed less predicted efficacy against planktonic and biofilm bacterial infections under standard clinical dosing. The variability in the simulated treatment outcome was only driven by interindividual PK variability. Accounting for the PD variability would require knowledge of the population distribution of key PD parameters (e.g., SlopeS, Kgs). Although clinical implications of these model-based predictions should be treated with caution, they could provide additional insights into the differences observed in preclinical *in vivo* or *in vitro* studies, for instance when evaluating and comparing treatment regimens.

Dose fractionation simulations identified ELF concentration-based fAUC/MIC and fCmax/MBIC for colistin, and fAUC/MIC and fAUC/MBIC for imipenem, as the PK/PD indices most predictive of treatment outcome. For colistin biofilm group, Emax was not reached within the evaluated dose range, which may lead to some uncertainty in the estimated maximal bacterial kill effect. However, the use of a sigmoidal Emax model does not introduce critical risks for PKPD index selection, as it is primarily based on the R^2^ values. A study using a neutropenic mouse model of biofilm lung infection identified serum fAUC/MIC and fAUC/MBIC as the best PK/PD indices of colistin for planktonic and biofilm infections, yet for imipenem f_T > MIC_ and f_T > MBIC_ were found to best predict the efficacy for planktonic cell and biofilm infections [53]. Plasma samples are more readily measurable than lung ELF concentrations in patients, thus, we examined the feasibility of using plasma concentration for the derivation of PK/PD indices. The plasma-based PK/PD indices were found to be as predictive as those derived from ELF concentrations. This consistency likely reflects the linear relationship between plasma and ELF exposure (i.e., Cmax and AUC) characterized by the population PK model [25,26]. As this analysis was based on simulations, further validation using clinical data is warranted.

Our current study has used static time kill studies of planktonic and biofilm bacteria to characterize the early phase antimicrobial PK/PD relationships. Experimental models utilizing dynamic continuous culture are of relevance to further characterize the response under prolonged antibiotic treatment conditions and generate more robust translational predictions towards humans. In addition, the 24 h-old alginate bead biofilm model may not fully reflect the heterogeneity and long-term dynamics of mature biofilms. Longer-term models, such as the CDC biofilm reactor, would therefore be relevant [54,55]. However, our recent study have found no difference in the minimal biofilm eradication concentrations of colistin between day 1 and day 6 [56], suggesting that efficacy evaluations using 24-h-old biofilms remain informative, despite its limitation.

This analysis did not consider the effects of immunity on antimicrobial PK/PD relationships. This aligns with the most common methods to derive PK/PD indices, i.e., in immune-suppressed mouse thigh infection models. In the clinical setting, we consider the lack of incorporating an immune-mediated killing component in our PK/PD simulations to represent a conservative estimate of expected bacterial PD effects. Besides, antimicrobial drug effects have been reported to be attenuated at high densities [57]. The impact of inoculum size could not be investigated in this study, mainly limited by the available experimental data.

Our analysis demonstrates how a semi-mechanistic pharmacodynamic modeling approach can facilitate further pharmacological interpretation of *in vitro* biofilm infection models, through the separation of drug- and biological system (e.g. planktonic or biofilm)-specific parameters, in line with related publications describing similar semi-mechanistic mathematical models reported for *in vitro* flow cell models and an *in vivo* rat lung infection model [[58], [59], [60]]. We expect that semi-mechanistic modeling approaches are relevant to allow the simultaneous integration of multiple pertinent pharmacodynamic readouts, e.g. CFU counts, biofilm mass, and metabolic activity.

In conclusion, we report a proof-of-concept analysis demonstrating the utility of mathematical pharmacodynamic modeling of *in vitro* biofilm time-kill assays and its integration with clinical PK models to explore translational predictions about antibiotic efficacy under biofilm-associated infections compared with planktonic infections.

## Funding

The research was financially supported by the 10.13039/501100004543China Scholarship Council.

## CRediT authorship contribution statement

**Yuchen Guo:** Formal analysis, Validation, Writing – original draft, Writing – review & editing. **Jinqiu Yin:** Formal analysis, Writing – original draft. **Linda B.S. Aulin:** Investigation, Methodology, Writing – review & editing. **Oana Ciofu:** Methodology, Resources, Writing – review & editing. **Claus Moser:** Methodology, Resources, Writing – review & editing. **Parth J. Upadhyay:** Investigation, Methodology, Writing – review & editing. **Niels Høiby:** Methodology, Resources, Writing – review & editing. **Hengzhuang Wang:** Methodology, Resources, Writing – review & editing. **Tingjie Guo:** Conceptualization, Formal analysis, Methodology, Supervision, Writing – review & editing. **J.G. Coen van Hasselt:** Conceptualization, Formal analysis, Methodology, Supervision, Writing – review & editing.

## Declaration of competing interest

The authors declare that they have no known competing financial interests or personal relationships that could have appeared to influence the work reported in this paper.

## Data Availability

Data will be made available on request.
