# Supplementary material for: Pharmacodynamic modeling of colistin and imipenem against in vitro Pseudomonas aeruginosa biofilms
Source: Biofilm. 2026 Aug 3;12:100387. doi: 10.1016/j.bioflm.2026.100387 (PMC13471946; doi:10.1016/j.bioflm.2026.100387)
Supplement: Supplementary file 1 [file mmc1.docx]

# SUPPLEMENTAL MATERIALS For

**Pharmacodynamic modeling of colistin and imipenem against *in vitro*** ***Pseudomonas aeruginosa* biofilms**

Yuchen Guo^1†^, Jinqiu Yin^1†^, Linda B.S. Aulin^1^, Oana Ciofu^3^, Claus Moser^2,3^, Parth J. Upadhyay^1^, Niels Høiby^2,3^ Hengzhuang Wang^3^, Tingjie Guo^1^, J. G. Coen van Hasselt^1*^

**Affiliations**

1. Leiden Academic Centre for Drug Research, Leiden University, Leiden, The Netherlands
2. Department of Clinical Microbiology, Rigshospitalet, Copenhagen, Denmark
3. Department of Immunology and Microbiology, Costerton Biofilm Center, Faculty of Health Sciences, University of Copenhagen, Copenhagen, Denmark

^*^ Corresponding author

^†^ These authors have contributed equally to this work

**Corresponding author:**

Coen van Hasselt

+31 71 5273266

[coen.vanhasselt@lacdr.leidenuniv.nl](mailto:coen.vanhasselt@lacdr.leidenuniv.nl)

**Table S1**. Overview of experimental design information used for the *in vitro* experiments with planktonic cultures and the alginate bead biofilm experiments.

| **Antibiotic** | **Phenotype** | **Samples** | **Timepoints(h)** | **Concentrations (mg/L)** | **MIC (mg/L)** | **MBIC (mg/L)** |
| --- | --- | --- | --- | --- | --- | --- |
| **Colistin^#^** | Planktonic | 126 | 0,1,2,4,8,12,24 | 0,1,4,16,64,256 | 2 | - |
|  | Biofilm | 126 | 1,2,4,8,12,24 | 0,1,4,16,64,256 | - | 8 |
| **Imipenem** | Planktonic | 168 | 0,1,2,4,8,12,24 | 0,0.5,1,2,4,8,16,32 | 1 | - |
|  | Biofilm | 168 | 0,1,2,4,8,12,24 | 0,0.5,2,8,32,128,512,2048 | - | 8 |

^#^ The concentrations of colistin in time-kill studies were demonstrated in milligram per liter (mg/L). Colistimethate sodium (CMS), used as a prodrug in clinical settings, is commonly represented in million international units (MIU). Unit conversion:1 MIU CMS = 80 mg CMS.

Abbreviations: MIC, minimal inhibitory concentration; MBIC, minimal biofilm inhibitory concentration

**Table S2.** Population characteristics underlying human PK models of colistin and imipenem.

| **Population** | **Characteristics** | **Median (min–max)** |
| --- | --- | --- |
| **Colistin population^a^** | Body weight (kg) | 81 (53–115) |
|  | Creatinine clearance (mL/min) | 122.5 (66–250) |
| **Imipenem population^b^** | Body weight (kg) | 85.3 (58.8–107.6) |
|  | Creatinine clearance (mL/min) | 133.3 (50.2–199.2) |

a, Colistin PK data from Boisson et al. (2014); b, Imipenem PK data from van Hasselt JGC et al. (2016).

**Figure S1.** **Raw observations for bacterial counts over time for colistin and imipenem in planktonic cultures and alginate-bead biofilm cultures.** Observations below the quantification limit (gray dashed line) were displayed as half of the quantification limit for illustration.

**Figure S2**. **Predicted antibiotic concentrations for colistin (upper) and imipenem (lower) during *in vitro* time-kill assays.** The parameters to account for colistin loss due to the binding with plastics and degradation were estimated based on the data from literature [1]. The degradation of imipenem was described using the average of degradation rate constants reported in the literature [2,3].

**Figure S3. Visual predictive check of the natural growth kinetics for planktonic bacteria (left) and biofilm bacteria (right).** The black points are the observed bacterial count data (Log10 CFU); the red lines represent the median values of model predictions; the blue areas are the prediction interval defined by of the 10th to 90th percentiles of the model predictions.

**Figure S4. Goodness-of-fit plots for the planktonic control growth model.** The upper panels show observed concentrations versus individual (left) and population (right) predictions. The lower panels show conditional weighted residuals (CWRES) versus population predictions (left) and time after dose (right). Solid lines represent the identity line or zero residuals, with dashed lines indicating ±1.96 CWRES limits.

**Figure S5. Goodness-of-fit plots for the biofilm control growth model.** The upper panels show observed concentrations versus individual (left) and population (right) predictions. The lower panels show conditional weighted residuals (CWRES) versus population predictions (left) and time after dose (right). Solid lines represent the identity line or zero residuals, with dashed lines indicating ±1.96 CWRES limits.

**Figure S6. Goodness-of-fit plots for the Colistin planktonic model.** The upper panels show observed concentrations versus individual (left) and population (right) predictions. The lower panels show conditional weighted residuals (CWRES) versus population predictions (left) and time after dose (right). Solid lines represent the identity line or zero residuals, with dashed lines indicating ±1.96 CWRES limits.

**Figure S7. Goodness-of-fit plots for the Colistin biofilm model.** The upper panels show observed concentrations versus individual (left) and population (right) predictions. The lower panels show conditional weighted residuals (CWRES) versus population predictions (left) and time after dose (right). Solid lines represent the identity line or zero residuals, with dashed lines indicating ±1.96 CWRES limits.

**Figure S8. Goodness-of-fit plots for the Imipenem planktonic model.** The upper panels show observed concentrations versus individual (left) and population (right) predictions. The lower panels show conditional weighted residuals (CWRES) versus population predictions (left) and time after dose (right). Solid lines represent the identity line or zero residuals, with dashed lines indicating ±1.96 CWRES limits.

**Figure S9. Goodness-of-fit plots for the Imipenem biofilm model.** The upper panels show observed concentrations versus individual (left) and population (right) predictions. The lower panels show conditional weighted residuals (CWRES) versus population predictions (left) and time after dose (right). Solid lines represent the identity line or zero residuals, with dashed lines indicating ±1.96 CWRES limits.

**Figure S10. Local parameter sensitivity analysis.** The colored tiles are the sensitivity value change of model output area under the bacterial growth curve (AUC_0-24h_) upon 10% increase in parameters for colistin planktonic model (A), colistin biofilm model (B), imipenem planktonic model(C) and imipenem biofilm model (D). The blue tiles represent a positive correlation between the parameter and model response, and the red tiles represent a negative correlation.

**Figure S11. Pharmacokinetic/pharmacodynamic (PK/PD) target analysis for colistin and imipenem against planktonic and biofilm infections based on plasma antibiotic concentrations.** Dose fractionation studies were simulated across a range of total daily doses and dosing intervals. The resulting plasma concentration-based PK/PD indices were correlated with the model-predicted change in bacterial density at 24 hours relative to baseline, with points representing each simulation and solid lines representing sigmoidal Emax model fits.

# *In vitro* PK modeling of colistin and imipenem in time-kill assay

**Methods**

Colistin and imipenem exhibit significant loss during *in vitro* experiments. To accurately capture PK/PD relationships, the concentration-time profiles of colistin and imipenem were reproduced while considering the degradation and binding kinetics during the time-kill assay (**Figure S2**).

The *in vitro* PK model for colistin was fitted using literature data [1]. The *in vitro* kinetics of colistin in time-kill assay involved binding to the polystyrene microwell plate (Nunc) surface and degradation over time. The binding kinetics between colistin and the binding sites (receptors) on Nunc plate consisted of (1) instantaneous irreversible binding, represented by bioavailability (*F1*), and (2) reversible binding, described by the association ($k_{on}$) and dissociation ($k_{off}$) rate constants for the colistin-receptor complex. The degradation of colistin was assumed to follow a first-order process, characterized by an elimination rate constant ($k$).

$$\frac{dC_{{drug}_{exp}}}{dt}={-k}_{on}\cdot C_{{drug}_{exp}}\cdot\left( M- C_{bind} \right)+k_{off}\cdot C_{bind}-k\cdot C_{{drug}_{exp}}$$

(**Eq.S1**)

$$\frac{dC_{bind}}{dt}= k_{on}\cdot C_{{drug}_{exp}}\cdot\left( M- C_{bind} \right)-k_{off}\cdot C_{bind}$$

(**Eq.S2**)

The *in vitro* PK of imipenem in the time-kill assay was primarily influenced by degradation, which was modeled using the first order elimination rate constant ($k$) averaged across studies [2, 3].

$$\frac{dC_{{drug}_{exp}}}{dt}= -k\cdot C_{{drug}_{exp}}$$

(**Eq.S3**)

**Table S3**. Parameters characterizing the *in vitro* loss of colistin and imipenem in the time-kill assay.

| **Antibiotic** | **Parameter** | **Description** | **Value (RSE%)** |
| --- | --- | --- | --- |
| **Colistin** | *F1* | Fraction of dose remaining unbound after instantaneous irreversible binding (bioavailability) | 0.836 (1) |
|  | $k_{on}$ | Association rate constant | 10.4 (47) |
|  | $k_{off}$ | Dissociation rate constant | 0.602 (144) |
|  | $M$ | Total concentration of reversible binding sites | 0.656 (19) |
|  | $k$ | First order elimination rate constant | 0.005 (22) |
| **Imipenem** | $k$ | First order elimination rate constant | 0.198^2,3^ |

RSE, relative standard error.

**Figure S12. Fitness of the literature *in vitro* pharmacokinetic data of colistin.** The *in vitro* pharmacokinetic of colistin was described with instantaneous irreversible binding, reversible binding and degradation. Points represent the observation data reported in Karvanen Matti et al (2017), and lines represent the prediction.

**References**

1.	Karvanen M, Malmberg C, Lagerbäck P, et al (2017) Colistin is extensively lost during standard in vitro experimental conditions. Antimicrobial Agents and Chemotherapy. https://doi.org/10.1128/AAC.00857-17

2.	Swanson DJ, DeAngelis C, Smith IL, Schentag JJ (1986) Degradation kinetics of imipenem in normal saline and in human serum. Antimicrobial Agents and Chemotherapy 29:936–937. https://doi.org/10.1128/aac.29.5.936

3.	White R, Friedrich L, Burgess D, et al (1996) Comparative in vitro pharmacodynamics of imipenem and meropenem against Pseudomonas aeruginosa. Antimicrobial Agents and Chemotherapy 40:904–908. https://doi.org/10.1128/aac.40.4.904
